# SCaFoS: a tool for Selection, Concatenation and Fusion of Sequences for phylogenomics

**DOI:** 10.1186/1471-2148-7-S1-S2

**Published:** 2007-02-08

**Authors:** Béatrice Roure, Naiara Rodriguez-Ezpeleta, Hervé Philippe

**Affiliations:** 1 Canadian Institute for Advanced Research, Centre Robert Cedergren, Département de biochimie, Université de Montréal, Montréal, Québec H3C3J7, Canada

## Abstract

**Background:**

Phylogenetic analyses based on datasets rich in both genes and species (phylogenomics) are becoming a standard approach to resolve evolutionary questions. However, several difficulties are associated with the assembly of large datasets, such as multiple copies of a gene per species (paralogous or xenologous genes), lack of some genes for a given species, or partial sequences. The use of undetected paralogous or xenologous genes in phylogenetic inference can lead to inaccurate results, and the use of partial sequences to a lack of resolution. A tool that selects sequences, species, and genes, while dealing with these issues, is needed in a phylogenomics context.

**Results:**

Here, we present SCaFoS, a tool that quickly assembles phylogenomic datasets containing maximal phylogenetic information while adjusting the amount of missing data in the selection of species, sequences and genes. Starting from individual sequence alignments, and using monophyletic groups defined by the user, SCaFoS creates chimeras with partial sequences, or selects, among multiple sequences, the orthologous and/or slowest evolving sequences. Once sequences representing each predefined monophyletic group have been selected, SCaFos retains genes according to the user's allowed level of missing data and generates files for super-matrix and super-tree analyses in several formats compatible with standard phylogenetic inference software. Because no clear-cut criteria exist for the sequence selection, a semi-automatic mode is available to accommodate user's expertise.

**Conclusion:**

SCaFos is able to deal with datasets of hundreds of species and genes, both at the amino acid or nucleotide level. It has a graphical interface and can be integrated in an automatic workflow. Moreover, SCaFoS is the first tool that integrates user's knowledge to select orthologous sequences, creates chimerical sequences to reduce missing data and selects genes according to their level of missing data. Finally, applying SCaFoS to different datasets, we show that the judicious selection of genes, species and sequences reduces tree reconstruction artefacts, especially if the dataset includes fast evolving species.

## Background

Phylogenomics, i.e. phylogenetic inference based on large amounts of sequence data, is an alternative approach to single gene phylogenies, which are insufficient to resolve many phylogenetic questions [1]. The most common phylogenomic strategies using primary sequences are the concatenation of sequences before tree reconstruction (super-matrix approach) and the combination of single gene phylogenies (super-tree approach). Several difficulties are associated with handling large amounts of data: (i) the uneven distribution of species across genes (genes that have been lost or that are not yet sequenced); (ii) the existence of partial sequences, especially in EST (Express Sequence Tag) and WGS (Whole Genome Shotgun)-based projects; and (iii) the presence of multiple copies per gene for the same species (paralogs or xenologs). The two first points imply the presence of missing data in the final dataset, whereas the third imply the presence of sequences that do not reflect the species tree and could therefore mislead phylogenetic inference.

Undoubtedly, one of the most problematic aspects when assembling phylogenomic datasets for reconstructing species trees is the presence of paralogous or xenologous genes. As opposed to orthologous genes, which arose by speciation and reflect the organismal phylogeny, paralogs arose by gene duplications, and xenologs, by lateral gene transfer from another species [2]. Both cases generally imply the presence of multiple copies of a given gene per species, some of which do not reflect the organismal phylogeny. Orthology assignment is a difficult task [3]. Similarity of primary sequence alone is not always sufficient to discriminate correct orthologs [4]. A rigorous, albeit extreme, solution would be to retain only genes having one and only one copy in all the species under study (an approach particularly suited when complete genomes are available). However, if an objective is to minimise the amount of missing data, this implies retaining a tiny fraction of the genome (e.g., 14 genes from 10 complete eukaryotic genomes in the study of Philip *et al*. [5]), rejecting a large number of genes whose paralogy history may be inferred and/or does not disturb the inference of the species phylogeny. In particular, this approach would uselessly reject in-paralogs (i.e. genes issued from a recent duplication within a single species), which do not disturb the inference of species phylogeny. In contrast, great care should be taken to detect out-paralogs (i.e. genes for which the duplication event arose before speciation) whose presence may induce erroneous phylogenies. Unfortunately, orthology determination is difficult when only one sequence per species exists. In brief, a gene should only be discarded when its duplication history cannot be reliably inferred meaning that gene and sequence selection should integrate information about duplication histories in order to optimally infer organismal phylogeny from genomic data.

Missing data are also often considered to be a significant obstacle in phylogenetic reconstruction (see Wiens 1998 [6] and references therein), and researchers generally prefer to avoid incomplete super-matrices [7,8]. Nevertheless, this implies that a compromise has to be made between using a large number of species for a few sequenced genes or a large number of genes for a few completely sequenced species. The first strategy often fails to provide statistically supported trees due to the limited sequence information contained in single or few genes, whereas the second can lead to highly supported, albeit erroneous trees, due to systematic biases (e.g. compositional or rate heterogeneity among lineages) [1,9]. Influence of systematic bias is limited with the first strategy because the impact of bias will be reduced as multiple substitutions (hence convergence) are detected more easily. Therefore, using a large number of both genes and species is necessary to infer accurate and well-resolved phylogenies, even if this implies the presence of missing data. To achieve this purpose, algorithms have been developed to identify optimal incomplete phylogenetic datasets [10,11] allowing the assembly of huge super-matrices (e.g. 70 taxa and 1131 genes [12]) automatically from a given database. However, this automation favours the selection of species for which the complete genome is sequenced, without consideration of their phylogenetic interest. For instance, it may lead to the inclusion of redundant taxa (e.g. mouse and rat when studying the eukaryotic phylogeny) or of rogue taxa (e.g. microsporidia), which would needlessly increase computational time and phylogenetic inaccuracy, respectively. Nevertheless, recent studies using simulations, as well as real data, have shown that the presence of missing data does not drastically reduce phylogenetic accuracy as long as a sufficient number of characters is available for each species [12-14]. That is the reason why reducing the amount of missing data must not be an end in itself. In particular, it has been shown that including partial sequences to break a long branch (i.e. adding species that are sister-group of a fast evolving species) reduce one of most common tree reconstruction artefacts, known as long branch attraction (LBA) [15]. In the same goal, an extreme approach is to exclude the fastest evolving genes from a fast evolving taxon (up to 90% of missing data for a given species) [16]. Even if these approaches imply much more incomplete matrices, the ultimate aim of selecting sequences, genes and species is to increase the amount of phylogenetic signal to the detriment of noisy signal; minimizing the level of missing data is one of the ways to pursue this aim. In fact, no rules currently exist to find the optimal number of taxa and level of missing data and a tool is therefore required to easily explore this question.

In summary, accurate and statistically supported phylogenetic inferences rely on the construction of large datasets with minimal amount of missing data and free of non-orthologous sequences, which makes species, gene and sequence sampling a crucial issue. In order to facilitate the construction of such phylogenomic datasets, we have developed SCaFoS, a tool that semi-automatically or automatically selects species, genes and sequences taking into account their level of missing data. Moreover, the software presents two novel functions: (i) it allows the combination of closely related species into a single pseudo-species to minimize missing data while retaining poorly represented taxa, and (ii) uses the relative evolutionary distance of the sequences and/or the user's expertise to judiciously select orthologous and/or slowest evolving sequences to avoid inaccurate phylogenetic reconstructions. These new functions will be peculiarly useful in a data mining context as more and more genomes will be sequenced.

## Implementation

SCaFoS runs in an easy-to-use graphical mode, as well as in a command-line mode that can be implemented in a workflow. It can deal with either amino acid or nucleotide sequences. Common formats for input and output alignment files are handled: Fasta, Phylip [17], Must [18] or Nexus [19]. SCaFoS is developed in Perl and the graphical interface is designed with Perl-Tk.

### Sequence selection

**Figure 1:**
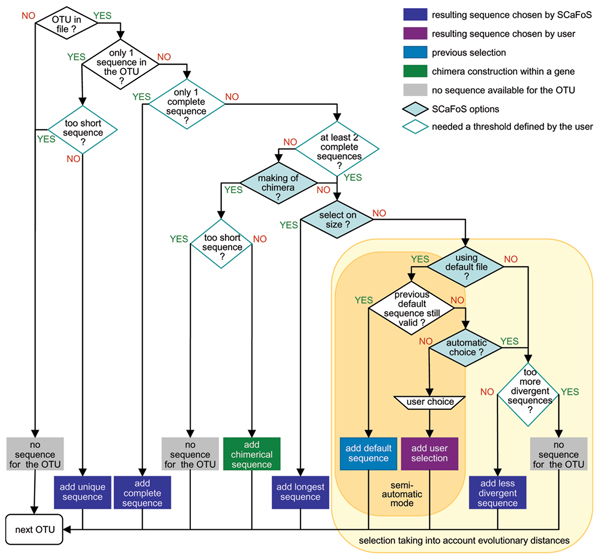
**Flowchart of sequences selection and construction of chimera for an OTU in a given gene**. For each OTU of each gene, SCaFoS selects the sequence that best represents the OTU. See text for a detailed description of the process. Three thresholds (empty blue rhombus) with default or user specific values are important: (i) the maximal percentage of characters present with respect to the longest sequence to keep a sequence, (ii) the minimal percentage of characters present with respect to the longest sequence to consider a sequence as complete and (iii) the maximum in-OTU/out-OTU distances ratio (see text) to keep an OTU. The user should select if he/she desires to create or not chimerical sequences and chose among the different sequence selection criteria (filled blue rhombus). If the selection criterion is the sequence size, no other options should be checked. If the selection criterion is the evolutionary rate of the sequences, the user must chose between a fully automatic or a semi-automatic choice of sequences and specify if he/she desires to use a previously defined selection.

The concept of Operational Taxonomic Unit (OTU) is an important aspect of SCaFoS. An OTU is a monophyletic group of species (possibly one) that will result, in the final dataset, into a single taxon labelled with the OTU name. Using a list of OTUs specified by the user, for each gene, SCaFoS will select the sequence that best represents a given OTU, ideally, the longest and slowest evolving orthologous sequence; evolutionary distance, as an approximation of the evolutionary rate, is estimated for each sequence. The sequence selection process for a single alignment file is summarized on a flowchart (Fig. 1) and described below. This crucial process is based on various thresholds defined in percentage of residues from the total number of positions (for the two first) or in percentage of the average evolutionary distance (for the last):

- the *minimum length *of a single sequence is used to remove too short sequences because stochastic errors might be induced by partial sequences, especially in the super-tree approach (default = 10%);

- the *sequence completeness *is defined to consider as complete a sequence for which few residues are missing (default = 10%),  called *quasi-complete *sequences;

- the *divergence threshold *is the maximum percentage of pairwise phylogenetic distance within the OTU compared to the average pairwise distances with the other sequences (default = 25%).

Schematically, the steps for sequence selection occur as follows according to the different thresholds:

- if only one sequence for a given OTU is present in the file, the sequence is systematically selected except if it is too short;

- if only one quasi-complete sequence (according to the sequence completeness criterion) exists for the OTU, the sequence is also systematically selected, even if this sequence has a higher evolutionary rate than the non-complete sequences in the OTU;

- if none of the sequences are quasi-complete and the chimera option has been chosen by the user, a chimerical sequence will be constructed and selected as described in 'Construction of chimerical sequences' paragraph, except if the created chimera is too short;

- if at least two quasi-complete sequences are present, only these quasi-complete sequences are sent to the selection criteria step described in 'Selection according to evolutionary distances' paragraph;

- otherwise, all incomplete sequences are sent to the selection criteria step.

Two mutually exclusive selection criteria, sequence size or evolutionary distances, constitute the starting point of the selection criteria step. The more straightforward criterion is the size of the sequences, in which case the longest sequence will be selected. Although this criterion is best to minimize the quantity of missing data, selection according to evolutionary distances allows a more judicious choice of sequences (see below). Those two kinds of sequence selection are provided in an automatic mode, which makes SCaFoS a stand-alone tool.

#### Selection according to evolutionary distances

For each gene alignment, evolutionary distances between each pair of sequences are calculated with TREE-PUZZLE [20]. While the choice of the model of substitution is left to TREE-PUZZLE, the user can enforce a Gamma distribution to handle rate heterogeneity across sites. In practice, we have observed that the assumption of uniform rates provides sufficiently accurate estimates, while significantly reducing computational time. Evolutionary distances are used in two goals: (i) verifying that the OTU does not include xenologous or paralogous sequences, and mainly (ii) selecting the least divergent sequence. Then, for each OTU, the ratio between the in-OTU distances (maximum pairwise phylogenetic distance within each OTU) and the out-OTU distances (the average pairwise distances between each OTU sequence and each non-OTU sequence) is calculated. If the in-OTU/out-OTU distances ratio is bigger than the divergence threshold, all sequences from this OTU will be discarded and, for this gene, the OTU will be represented by question marks in the super-matrix. Otherwise, the sequence that displays the lowest average distance to the other sequences will represent the OTU. This approach is rather drastic, but it is efficient to avoid out-paralogs in the resulting file. Nevertheless, as detailed below, a more accurate selection might be obtained with the semi-automatic mode. Evidence of gene duplication somewhere in the tree is a reason to worry about the orthology of the other sequences; then a more conservative option is also available which eliminates the complete gene when at least one OTU needs to be removed.

Finally if the OTU does not present risk of xeno- or paralogy, the less divergent sequence is selected from the quasi-complete sequences of the OTU in order to decrease the noisy signal contained in the terminal branches file (without decreasing the phylogenetic signal contained in the inner branch). For this last step, the definition of the sequence completeness is an important option because it is useful to be able to select an almost complete slow divergent sequence than a complete but highly divergent one.

#### Selection according to user's expertise

In the semi-automatic mode, after computation of the ratio in-OTU/out-OTU distances as previously described, SCaFoS proposes the user to select of the sequence that displays the lowest average distance. A visual flag indicates if the ratio in-OTU/out-OTU distances overcomes the user defined divergence threshold. In this manner, the user can choose between selecting the suggested sequence, or another complete sequence that he/she considers of better orthology, or discarding the OTU from this gene. The user can use any external information to validate his/her choice, in particular a phylogenetic tree or the position of the genes on the chromosome (synteny). The use of human expertise is advised because there are no known reliable methods for automatically identifying orthologs. As this user intervention is time consuming, SCaFoS saves the information on selected sequences. In subsequent dataset constructions, this information can be reused allowing for a fast assembling of numerous combinations of genes and taxa. The sequence selected in the first run for each OTU becomes the default sequence for a given OTU. As long as the list of complete sequences included in the OTU remains unchanged (i.e. no sequence are added or removed), SCaFoS automatically keeps the default sequence.

#### Construction of chimerical sequences

**Figure 2:**
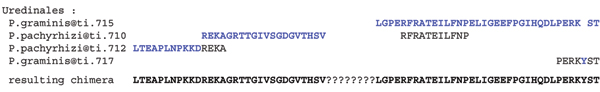
**Example of chimera assembly**. Sequence fragments are combined from longest to shortest, the length being computed according to the number of characters: selected parts are displayed in blue; the chimerical sequence is the result of the concatenation of each part of the different sequences

When an OTU lacks a complete sequence, creating a chimera within a gene may be a judicious choice to decrease the amount of missing data and the inclusion of species with few sequenced genes. A chimera is created from several partial sequences belonging to a particular monophyletic group. Sequences are incorporated into the chimerical sequence in descending order of sequence length as shown on Figure 2. Only the length of the sequences determines the order of incorporation of fragments in the chimera; if some partial sequences overlap, the fragment kept is the first incorporated.

Finally, SCaFoS is able to modulate between the creation of chimera from partial sequences and the selection of complete sequences, by considering sequences with few missing characters as full-length sequences.

### Global level of missing data

Once the sequences are selected for each gene, the user may want to select genes according to their global level of missing data. For this purpose, SCaFoS creates several directories that contain the processed files including the selected species and sequences. These files are sorted according to their level of missing species or characters and an additional file, containing the super-matrix is also produced for each level. Since there are no established rules on the maximum amount of missing data in a super-matrix, the user is free to select the threshold of missing data (either globally or for the species of interest) that he/she considers appropriate. For this purpose, the user is guided by the statistical information about the composition in genes, species and missing positions, the nature of phylogenetic question being also of major importance.

## Results and Discussion

### Typical use of SCaFoS

**Figure 3:**
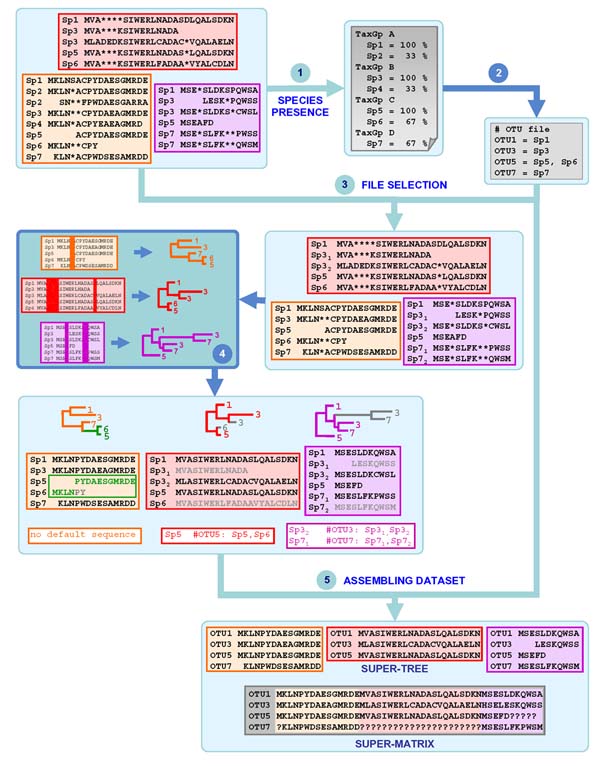
**Main steps to use SCaFoS**. Steps 1, 3 and 5 are done by SCaFoS: 1. SPECIES PRESENCE: listing of all species present in the files of aligned sequences followed by their frequency of presence and, if desired, classified into taxonomic groups (specified by TaxGp in the figure). 2. Definition by the user of the species to be selected and their respective OTUs 3. FILE SELECTION: creation of files containing only the selected species 4. Discarding ambiguously aligned positions (displayed in dark colour) with a tool such as GBlocks [33]; making phylogenetic trees (using PHYML [34] or PAUP [25] for example) 5. DATASETS ASSEMBLING: selection of sequences and chimera construction according to an OTU file and default sequence files: creation of single gene files including chimeras and selected sequences and creation of concatenated files for super-tree and super-matrix approaches respectively. In the last step, three typical cases are represented: (i) construction of a chimera (OTU5) in the orange file, (ii) selection of the less divergent sequence within an OTU (Sp6 in OTU5) and elimination of a short sequence (Sp3_1_) in the red file and (iii) elimination of potential paralogous sequences by the user (Sp3_1 _and Sp7_1_) in the purple file. Eliminated sequences are drawn in grey. The corresponding default sequences files are displayed under their respective sequence files.

Starting from files of aligned sequences, SCaFoS proceeds in three major steps (see Fig. 3 for an overview, and [1,9,16,21-23] for examples). First, it provides a file in which the species are sorted according to their frequency, i.e. average representation across genes, and taxonomic affiliation (Fig. 3, step 1: SPECIES PRESENCE). This file can then be used by the user as a guide to select organisms (species or strains) and define OTUs (Fig. 3, step 2) that would be used to construct chimerical sequences.

Second, using the OTUs defined by the user, SCaFoS creates a copy of each file that will contain only the sequences of the species of interest. It should be noted that no chimerical sequences will be created at this step, and all sequences from a given OTU will be included in each file (Fig. 3, step 3: FILE SELECTION). With a reduced number of sequences, one can more accurately remove ambiguously aligned positions in each file, and construct preliminary phylogenetic trees of each gene to control for laterally transferred or paralogous genes (Fig. 3, step 4).

Third, for each OTU and each gene, SCaFoS selects one sequence or constructs a chimerical sequence following the steps shown on Figure 1, and assembles final datasets (Fig. 3, step 5: ASSEMBLING DATASETS). In the semi-automatic mode, the user incorporates information from the trees constructed for single-genes (step 4) to select sequences. Moreover, if phylogenetic trees are available in postscript format (produced by MUST [18]), the selection is visually reported onto the trees.

Finally, all the relevant information about sequence selection is provided in a text file, allowing the analysis to be reproduced. Once the sequences are selected for each gene, files for super-matrix and super-tree analyses are generated in formats usable by MrBayes [24], PAUP [25], PHYLIP [17], or TREE-PUZZLE [20]. Files summarizing the presence of OTUs for each gene and the amount of missing data in various datasets help the user to select the best set of genes for subsequent inferences.

### Evaluation of SCaFoS performance

#### Impact of missing data

**Figure 4:**
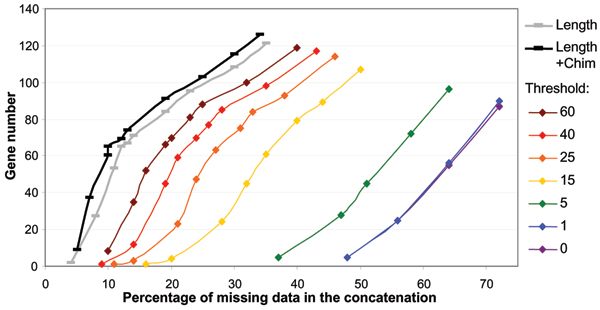
**Evolution of missing data according to the threshold**. For seven threshold values defining the maximal in-OTU/out-OTU distances ratio, the number of selected genes is plotted against the percentage of missing sites in the concatenated file. Subsets are extracted from the Metazoa dataset without making of chimera. The evolution of missing data is also displayed when the selection is only made according to the size criterion (black and grey curves respectively with and without making of chimera); these last selections represent the minimal amount of missing data for the dataset.

To evaluate the effect of our sequence selection approach on the level of missing data, we performed several analyses with different criteria: (i) selection of the longest sequence with and without the creation of chimeras, and (ii) without creation of chimeras, selection of the longest versus the slowest evolving sequence as long as the in-OTU distance is below a given threshold of the in-OTU/out-OTU distance ratio (between 0 and 60 percent, see above). We used the Metazoa dataset of 161 proteins from 49 animal and fungal species from Philippe et al. [22]. Similar results were obtained with the dataset of 169 nuclear aligned sequence files from 34 eukaryotes used by Rodriguez-Ezpeleta *et al*. [23], even if the differences are less important (data not shown). The statistics files produced by SCaFoS allow an easy monitoring of the missing data level according to these criteria (Fig. 4).

First, the use of chimerical sequences slightly reduces the level of missing data. For instance, for a global level of 30% of missing data, chimeras allow the incorporation of seven additional genes (115 versus 108). This is not surprising because the Metazoa dataset is mainly constructed from EST sequences, implying that data will frequently be missing for the same, lowly expressed genes. In practice, chimeras are especially interesting for OTUs having a key phylogenetic position (i.e. that break long branches or that are the only representative from a taxonomic group of interest).

Second, the conservative elimination of sequences when several copies are present for a given OTU, as performed in the automatic mode of SCaFoS, has much more drastic consequence. When the ratio in-OTU/out-OTU distances is 60%, 25%, or 1%, the global percent of missing data in the final dataset is 16, 24 and 64, respectively. Nevertheless, a similar number of genes (52, 47 and 56, respectively) is incorporated in the datasets. Note that this severe effect is not only due to paralogy, but is an incidental consequence of chimera construction through the OTU concept. In fact, when an OTU contains several species, the orthologous copies from these species are artificially considered in the exact same way as paralogs from the same organisms. Then, the more divergent species within the OTU are, the more likely SCaFoS will remove the OTU because at least one sequence will have a higher evolutionary distance than permitted by the divergence threshold. In such case, the automatic approach of SCaFoS is too conservative. We strongly recommend the use of the semi-automatic mode in which sequences are discarded only when paralogy problems are recognized by the user. Nevertheless, the automatic mode yields reasonable results when each OTU is represented by a single species (data not shown).

#### Sequence selection and the reduction of tree reconstruction artefacts

An important function of SCaFoS is to automatically determine, for each OTU, the best sequence for representing a given gene according to user-defined criteria. When several complete sequences are present for an OTU, SCaFoS tries to select the one that possesses the maximum amount of phylogenetic signal. To achieve this, the sequence that has the lowest evolutionary distance to all other sequences is selected to represent the OTU. As we will show, this approach helps to reduce the long branch attraction (LBA) artefact [26].

**Figure 5:**
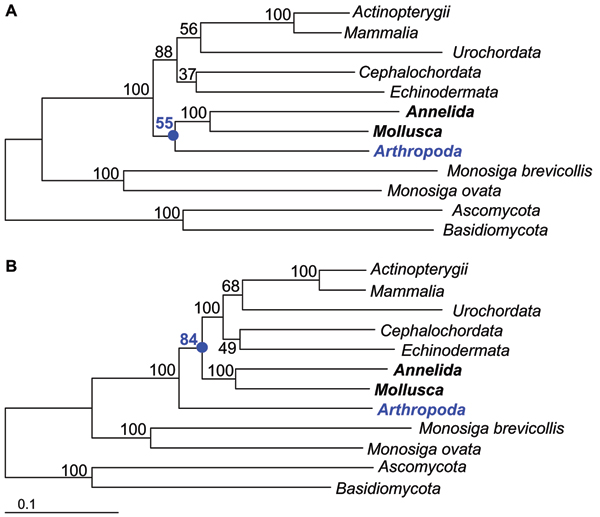
**Phylogenetic trees obtained for three subsets extracted from the Metazoa dataset**. Maximum Likelihood inferences were performed with the JTT+Γ (4 categories) model by TreeFinder [27] on two datasets based on the Philippe et al. [22] Metazoa dataset and constructed as follows. The species were grouped according to 12 OTUs. Sequences with at least 90% of the total number of positions were considered as complete and sequences or chimera shorter than 10% of the total number of positions were removed. The two datasets differ on the main criteria of selection, A: longest sequence (LC) and B: smaller evolutionary distances (SC). Numbers above branches indicate bootstrap support values obtained by analysing 100 bootstrap replicates under the same conditions.

Based on the Metazoa dataset, two super-matrices were automatically constructed using two different criteria of selection within an OTU, all the other options being left to defaults: (i) selection of the longest sequence (LC) among all sequences respecting the completeness criteria, and (ii) selection of the quasi-complete sequence with the smallest estimated evolutionary distance (SC) (with respect of completeness, the longest sequence is selected only when several sequences are equally least divergent). In both cases, chimeras were created when no quasi-complete sequences were available. Twelve OTUs covering the diversity of opisthokonts (animals + fungi) were considered. We analysed the concatenations of 140 proteins, which is similar to the number used in the original paper (146), where SCaFoS had been used in a semi-automatic mode [22]. The two datasets contained 32,648 unambiguously aligned amino acids with about 23% of missing data (corresponding to OTUs that lack sequence for some genes, this lacks being similar in the two datasets). Phylogenies were inferred by Maximum Likelihood with TreeFinder [27], using the JTT matrix of amino acid substitution [28] with a gamma distribution to correct rate across sites variation (JTT+Γ) model. With the SC concatenation, arthropods are sister-group of Lophotrochozoa (molluscs + annelids), recovering the expected monophyly of protostomes (Fig. 5A). In contrast, the phylogeny based on the LC concatenation recovers an erroneous bilaterian phylogeny, with deuterostomes grouped with Lophotrochozoa to the exclusion of arthropods (Fig. 5B). Importantly, the erroneous tree receives a higher support than the correct one (84% versus 55% bootstrap support). The explanation is simply that, in the LC super-matrix, arthropods are often represented by *Drosophila melanogaster *(95% versus 11%, respectively for LC and SC, see table 1), for which the complete genome sequence is available, but which evolves rather fast. As a result, arthropods are strongly, yet artefactually, attracted by the long branch of the outgroup. However, in the SC dataset, arthropods are represented by a mix of sequences of *Drosophila *and other slower evolving species when the latter have quasi-complete sequences, decreasing the global relative evolutionary distance of the OTU in the dataset. This example also illustrates the importance of the completeness option.

**Figure 6:**
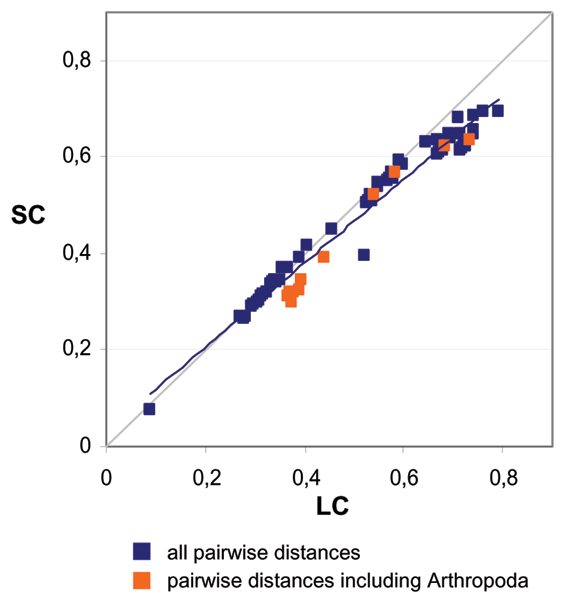
**Comparison of evolutionary distances**. The datasets are the same as in Figure 5. The phylogenetic inferences were obtained as for Figure 5. Pairwises of patristic distances are plotted in blue (dots including Arthropoda in orange).

However, the branch length of arthropods does not appear significantly longer on Figure 5B than on Figure 5A. We therefore directly compared the evolutionary distances between all pairs of species for the SC and LC concatenations using the same model (JTT+Γ). As expected, the LC distances are always larger than the SC distances (Fig. 6). This is particularly true for arthropods (orange squares), in agreement with our hypothesis of an LBA artefact affecting the result on Figure 5B. This didactical example illustrates that reducing the global amount of missing data (i.e. selecting the longest sequences) as a unique selection criterion can be misleading. The various criteria proposed by SCaFoS (in particular, the lowest evolutionary distance) allowed increasing the phylogenetic signal in the super-matrix, efficiently reducing the negative effect of LBA (Fig. 5).

### Importance of the investigator expertise

**Figure 7:**
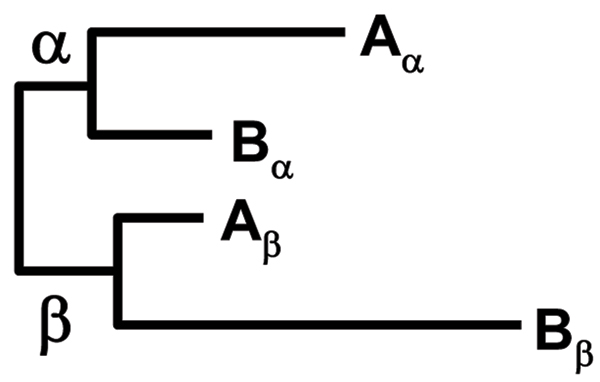
**Difficulty to determine correct orthologs according to the evolutionary distance**. Schematic tree representing two paralogous groups, α and β, including the same species, A and B. In this example, the choice of the two slowest evolving sequences, A_β _and B_α_, will keep a sequence in each paralogous group.

Although the automatic approach of SCaFoS is rather crude, the resulting datasets can be used for preliminary analyses (e.g. Fig. 5A). Yet, to build a final dataset, the semi-automatic approach should be preferred. In this mode, when the choice among multiple sequences for an OTU is ambiguous, the software guides the user by providing the average evolutionary distances (to reduce LBA) as well as missing data information. Moreover, to reduce compositional bias, another source of tree reconstruction artefact [29], the global deviation of amino acid or nucleotide composition is displayed as a complementary guide. For each sequence, the compositional deviation is computed as the sum of the deviation per residue between the current sequence and the whole sequence file. However, the latter information is not taken into account by SCaFoS to perform its selection. In complement, the use of a phylogenetic tree for each gene, inferred during step 4 of the proposed methodology (Fig. 3), is recommended for the selection of orthologs. In fact, the relative evolutionary distance of the sequences is not always a sufficient criterion, as exemplified on Figure 7, where the two slowest sequences (B_α _and A_β_) are paralogous sequences for species A and B. For all these reasons, we highly recommend to use SCaFoS in the semi-automatic mode.

Since there is no clearly defined limit for an acceptable level of global missing data, the investigator is free to choose his/her favourite compromise between the number of genes, the frequency of missing data and the severity of the threshold used to extract the orthologs. To do that, the user is guided by a table containing the number of genes, of positions and of missing data for each subdirectory in which the resulting files with a given amount of missing data have been copied.

### Perspectives

Some improvements could be considered. The most evident one is to take into account compositional biases when selecting sequences, especially when several sequences within an OTU have similar relative evolutionary distances. However, combining this criterion with the evolutionary distance is not straightforward because the compositional bias is not always correlated with the evolutionary distance. As we have shown, the sequence length is not the best criterion to choose a sequence and estimating the evolutionary distances of partial sequences to create intra-gene chimeras would improve the results. Yet, the evolutionary distance of each fragment should be corrected for the difference in the average evolutionary rate of this protein part because a conserved domain of a fast evolving species may have a slower evolutionary rate than a variable domain in a less divergent species. Taken into account the evolutionary distance for chimera making has also two advantages (i) avoiding risk of artificial heterotachy (i.e. incorporating partial sequences with various evolutionary rates), (ii) allowing the comparison of complete and chimerical sequences to select the slow evolving one. An idea to create chimera might be to infer ancestral state for each site; unfortunately, this rule is difficult to apply because it needs a within OTU phylogenetic tree and at least 4 residues per site, two conditions rarely met when few overlapped sequences like those obtained by EST methods are considered. Finally, incorporating refined tools to facilitate species selection (i.e. the definition of the OTUs), such as the biclique and quasi-biclique algorithms [10,11] would be also useful.

## Conclusion

Phylogenetic studies based on a huge sampling of both genes and species remain rare despite the great quantity of genomic data currently available. We have conceived a software open to a large usage in a phylogenomic context. SCaFoS is a helpful tool for rapidly constructing large datasets of aligned sequences that can be easily used with different phylogenetic inference approaches. Simplifying the construction of these datasets should permit a better phylogenetic use of genomic data by various samplings of sequences, species and genes. This latter point is particularly important because of the increasing number of contradictory papers that are based on different samples, as illustrated by the question of Ecdysozoa monophyly [5,22,30-32]. Finally, we have shown that SCaFoS selection of the slowest evolving representative sequence of a monophyletic group is an efficient approach to reduce the impact of tree reconstruction artefacts, suggesting that increasing the amount of phylogenetic signal during the construction of phylogenomic datasets should be a priority for future research.

## Availability and requirements

Project name: SCaFoS

Project home page: 

Operating systems: native Xwindow environment on Unix/Linux, Mac OSX and Windows platforms (Win32)

Programming language: Perl version 5.8.0 or later

Other requirements: Tcl/Tk version 8.4.5 or later and Tree-puzzle version 5.1 or later

## List of abbreviations

EST: Expressed Sequence Tags

LBA: Long Branch Attraction

OTU: Operational Taxonomic Unit

WGS: Whole Genome Shotgun

## Authors' contributions

HP and BR conceived the software. BR realized all the development and drafted this manuscript and the user manual. NRE and HP performed software testing and helped in writing the user manual. All the authors are involved in the final manuscript.

## Figures and Tables

**Table 1:** Selection frequency for species included in the Arthropoda OTU, Number of sequences per species and their corresponding frequency in the two datasets used for Figures 5 and 6 and constructed according to two different selection criteria: longest sequence (LC) or smallest evolutionary distance (SC)

	LC		SC	
	
	number of sequences	frequency	number of sequences	frequency
	
*Drosophila melanogaster*	133	95%	16	11%
*Anopheles gambiae*	3	2%	34	24%
*Bombyx mori*	1	1%	12	9%
*Litopenaeus vannamei*	1	1%	2	1%
*Hypsibius dujardini*	1	1%	3	2%
*Myzus persicae*	1	1%		
*Tribolium castaneum*			11	8%
*Apis mellifera*			9	6%
*Spodoptera frugiperda*			8	6%
*Amblyomma americanum*			7	5%
*Ctenocephalides felis*			7	5%
*Mesobuthus gibbosus*			6	4%
*Ornithodoros porcinus*			5	4%
*Manduca sexta*			4	3%
*Glossina morsitans*			3	2%
*Toxoptera citricida*			3	2%
*Callosobruchus maculatus*			3	2%
*Curculio glandium*			2	1%
*Acyrthosiphon pisum*			1	1%
*Ips pini*			1	1%
*Biphyllus lunatus*			1	1%
*Dermacentor variabilis*			1	1%
*Clytus arietis*			1	1%
